# The expanding genetic and clinical landscape associated with Meier-Gorlin syndrome

**DOI:** 10.1038/s41431-023-01359-z

**Published:** 2023-04-14

**Authors:** Emily Nielsen-Dandoroff, Mischa S. G. Ruegg, Louise S. Bicknell

**Affiliations:** Department of Biochemistry, University of Otago, Dunedin, New Zealand

**Keywords:** Disease genetics, Medical genetics

## Abstract

High-throughput sequencing has become a standard first-tier approach for both diagnostics and research-based genetic testing. Consequently, this hypothesis-free testing manner has revealed the true breadth of clinical features for many established genetic disorders, including Meier-Gorlin syndrome (MGORS). Previously known as ear-patella short stature syndrome, MGORS is characterized by growth delay, microtia, and patella hypo/aplasia, as well as genital abnormalities, and breast agenesis in females. Following the initial identification of genetic causes in 2011, a total of 13 genes have been identified to date associated with MGORS. In this review, we summarise the genetic and clinical findings of each gene associated with MGORS and highlight molecular insights that have been made through studying patient variants. We note interesting observations arising across this group of genes as the number of patients has increased, such as the unusually high number of synonymous variants affecting splicing in *CDC45* and a subgroup of genes that also cause craniosynostosis. We focus on the complicated molecular genetics for *DONSON*, where we examine potential genotype-phenotype patterns using the first 3D structural model of DONSON. The canonical role of all proteins associated with MGORS are involved in different stages of DNA replication and in addition to summarising how patient variants impact on this process, we discuss the potential contribution of non-canonical roles of these proteins to the pathophysiology of MGORS.

## Introduction

Meier-Gorlin syndrome (MGORS) is a rare form of microcephalic primordial dwarfism (MPD) with less than 100 cases reported in the literature. MPD is an umbrella term for a group of rare disorders defined by global growth restriction including microcephaly. The reduced head size is often proportional to the height reduction but can be more severe for some subcategories of MPD, such as Seckel syndrome. Generally, these disorders are inherited in an autosomal recessive manner. Associated genes encode proteins involved in processes required for timely cell cycle progression such as cell cycle checkpoint, centrosome duplication and coordination, DNA replication, and DNA repair [1]. The molecular pathophysiology suggests reduced cellular proliferation (or increased apoptosis) during development ultimately underlies the restricted growth [1]. MGORS is diagnosed clinically by the triad of short stature, microtia, and patella hypo/aplasia, although not all MGORS patients present with all three (Table 1) [2]. There is a common facial gestalt of downslanting palpebral fissures, a fuller bottom lip, and with age, the nose becomes more prominent. Additional features are variably present between individuals (Table 1), with the exception of mammary hypoplasia which is completely penetrant in post-pubescent females [2].

**Table 1:** Frequency of features in individuals with a clinical and genetic diagnosis of Meier-Gorlin syndrome.

Information was derived from the reported cases in the literature and clinical information provided, so the number of cases in the literature likely underestimates the true number of affected individuals, especially for established disease genes. *na* information not available. *IUGR* intra-uterine growth retardation, *ID* intellectual disability, *DD* developmental delay.

MGORS is caused by disruption to DNA replication and is typically associated with early DNA replication processes [3–12]. DNA replication is a complex multi-protein process that is tightly regulated to ensure accurate duplication of the genome (reviewed in refs. [13, 14]). The initiation of DNA replication involves the formation of the pre-replication complex (pre-RC). This occurs during the M/G1 phases of the cell cycle and is initiated by localisation of the origin recognition complex (ORC, composed of subunits ORC1-6) to origins of replication. Recruitment of CDC6 and interaction with CDT1 facilitates the stepwise addition of two inactive MCM2-7 hexamers (MCM) onto chromatin in a process called replication origin licensing. Inhibition of CDT1 by GMNN during S and G2 phases ensures that this licensing step only occurs once during each cell cycle. Origin activation occurs during the S phase transition and first requires the formation of the pre-initiation complex (pre-IC). The pre-IC unites multiple replication factors to chromatin-bound MCM, including CDC45 and the GINS complex (GINS1-4), which associate together with MCM to form the CMG complex. The CMG complex facilitates the activation of MCM helicase activity, which is responsible for unwinding double-stranded DNA during S-phase. Further replication factors are then recruited to form the replisome, a large multi-protein complex that couples the unwinding of double-stranded DNA with active DNA synthesis by DNA polymerases. Multiple progression and cell-cycle checkpoint proteins, including the replication fork stabilisation factor DONSON [13, 15], function at the replisome to maintain genome integrity by ensuring continued and efficient progression of the replisome.

**Fig. 1:**
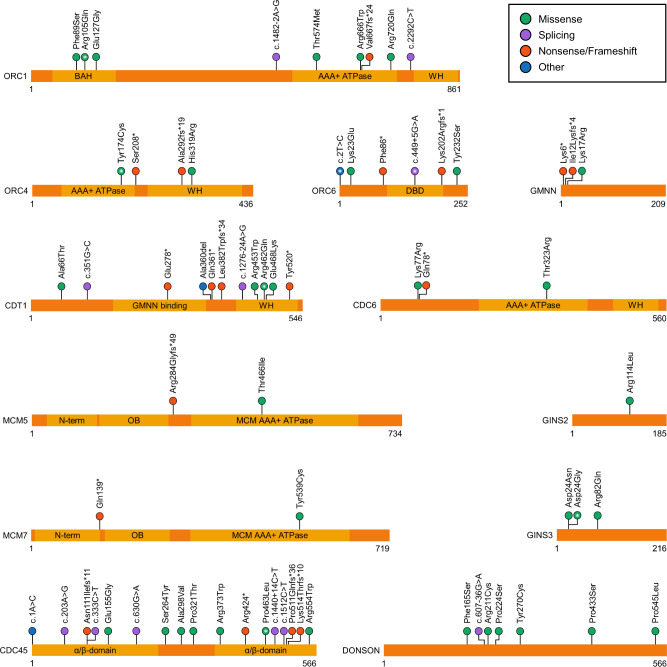
Meier-Gorlin syndrome variants and their locations. Schematics for each MGORS protein detailing published MGORS variants and known protein domains. Circles containing a white asterisk (*) represent variants that are recurrent in three or more families. Domain name abbreviations: BAH (bromo-adjacent homology), WH (winged helix), DBD (DNA binding domain), OB (oligonucleotide/oligosaccharide binding). RefSeq IDs: ORC1 (NM_004153.3), ORC4 (NM_181741.3), ORC6 (NM_014321.3), GMNN (NM_015895.4), CDT1 (NM_030928.3), CDC6 (NM_001254.3), MCM5 (NM_006739.3), MCM7 (NM_005916.4), GINS2 (NM_016095.2), GINS3 (NM_022770.3), CDC45 (NM_003504.4), DONSON (NM_017613.3).

Variants in 13 DNA replication genes have been associated with MGORS, these are *ORC1*, *ORC4*, *ORC6*, *CDT1, CDC6, GMNN, MCM3*, *MCM5*, *MCM7, CDC45, GINS2*, *GINS3*, and *DONSON* (Fig. 1) [3–12]. Twelve of these have essential roles in early DNA replication, including nine which are specifically involved in pre-RC formation. Consequently, MGORS has been defined by the reduced ability to load early replicative machinery onto replication origins [13]. The identification of replication fork stabiliser *DONSON* as a new MGORS gene challenges this idea [9, 16, 17], since DONSON has no known role during early DNA replication processes.

The use of hypothesis-free high throughput sequencing methods, alongside an increase in patient cohorts, continues to expand both the genetic and phenotypic spectrums of MGORS. This review presents an overview of the current MGORS-associated genes, focusing on molecular causes and clinical presentations for each gene.

## Genes contributing to Meier-Gorlin syndrome

### ORC1

The first individuals identified with biallelic variants in *ORC1* have a generalised primordial dwarfism [4]. Based on the serendipitous finding of more severe *ORC1* variants in a child with a complex congenital disorder with features reminiscent of MGORS, further sequencing efforts led to the discovery that variants in *ORC1* are a common cause of MGORS, including the initial patient described by Gorlin [3, 18].

*ORC1* individuals have characteristic MGORS features, though not all have the complete triad (Table 1) [19]. These individuals also show a spectrum of additional rarer phenotypes, including a range of neurological, respiratory, cardiac, gastrointestinal, urogenital, and musculoskeletal abnormalities [19]. This spectrum is relatively consistent with the MGORS presentations caused by other genes, however, *ORC1* individuals have significantly more severe growth restrictions in height and head circumference [19, 20]. One individual has craniosynostosis [19], a feature typically seen in MGORS caused by variants in *CDC45*. Lethality is rare in MGORS but has been observed in a family segregating a frameshift variant together with the recurrent p.Arg105Gln variant [3], suggesting more severe variants could be linked to more severe outcomes in affected individuals. In considering the two originally described cohorts of individuals with variants in *ORC1* (the generalised primordial dwarfism versus MGORS), individuals with MGORS tend to have at least one variant that is predicted to lower ORC1 protein levels, such as splicing, frameshift, or genomic deletions [3]. While not universal [7], this could point towards a pathological mechanism for why some patients have the additional specific features of MGORS that are absent from the primordial dwarfism cohort.

Many of the patients with *ORC1*-associated MGORS have substitutions in the N-terminal bromo adjacent homology (BAH) domain (Fig. 1), which is important for interactions between ORC and both chromatin-bound histones and DNA [21–23]. The most commonly reported MGORS ORC1 substitution, p.Arg105Gln, is located within this BAH domain and impairs the essential ORC function of chromatin binding [4, 23]. While there is variant clustering, missense variants affecting ORC1 are not solely confined to the BAH domain, for example, the p.Arg720Gln substitution is located within the ATPase domain and has a disruptive effect on ATP hydrolysis, which is essential for complex assembly on chromatin (Fig. 1) [24]. Other variants have splicing effects, decrease protein stability, or result in protein truncation (Fig. 1) [3]. The commonality amongst these variants appears to be the impaired rate of cell cycle progression [4].

Analysis of zebrafish models generated through morpholinos targeting *orc1* showed significant decrease in overall growth, consistent with the MGORS phenotype [4]. Co-injection of morpholino and *ORC1* mRNA demonstrated that this growth-restricted phenotype could be rescued by wildtype *ORC1*, but not *ORC1* mRNA containing amino acid substitutions that impaired histone binding [21].

### ORC4

Variants in *ORC4* were identified independently by two groups in the first tranche of genes associated with MGORS [3, 7]. For *ORC4*, the most commonly reported causal variant is p.Tyr174Cys, which has been inherited in either a homozygous state or as a compound heterozygous variant inherited with nonsense variants [3, 7]. Tyr174 is located in the AAA + ATPase domain of ORC4 and is completely conserved throughout eukaryotes (Fig. 1) [3, 7]. Tyr174 is an important residue for ATPase activity, with activity decreased by 50% when the tyrosine is substituted to cysteine [24]. The p.Tyr174Cys change likely impairs the essential role of ORC4 ATP hydrolysis in facilitating ORC formation [7, 25]. Modelling of this variant in yeast caused reduced progression through S phase which impacted organism proliferation rates [7].

A *Drosophila* model studying the recurrent p.Tyr174Cys variant (p.Tyr162Cys in *Drosophila*) reported reduced viability in the homozygous state, along with tissue-specific effects in bristle spacing and length [26]. Similar bristle effects have also been observed in *orc6 Drosophila* mutants [27]. Females were sterile, likely due to decreased EdU incorporation affecting endoreplication, ultimately impacting chorion gene amplification.

Individuals with *ORC4* variants generally have the clinical triad characteristic for MGORS and have the second most severe growth defects behind *ORC1*-associated individuals (Table 1) [20]. There also appears to be a genotype-phenotype correlation, as more severe features are present in individuals harbouring one loss-of-function variant in trans with a missense variant compared to those individuals with two missense variants [19]. Aligning with this, those cases with a predicted more severe genotype also commonly displayed additional clinical features, such as pulmonary emphysema (1/3 cases) or the need for tube feeding (2/3 cases) [19].

### ORC6

Variants in *ORC6* were also identified in the first tranche of genetic discoveries for MGORS [3]. *ORC6* was screened as a candidate gene based on the role of the encoded protein as an ORC subunit, but this interaction appears to be more dynamic than other ORC6 subunits in different species, with human ORC6 being more loosely associated with the other ORC subunits [28]. Rather than being an integral subunit of ORC for binding to DNA, ORC6 appears to be more important in protein-protein interactions, such as with CDC6 [29]. Despite this, MGORS variants in ORC6 disrupt the ORC complex function in a similar fashion to other ORC variants.

Much like cases with *ORC1* or *ORC4* variants, not all individuals with *ORC6-*associated MGORS have all three of the characteristic features (10/11 cases with short stature, 7/8 cases with patellar hypo/aplasia) (Table 1) [19]. While most ORC6 MGORS patients have a relatively mild phenotype, one family is an exception, with a homozygous c.602_605delAGAA, p.Lys202Argfs*1, variant causing very severe growth and developmental defects in three terminated pregnancies [30]. Approximately one-third of reported individuals with causal variants in *ORC6* have the biallelic missense variants c.2T>C and c.449+5G>A [19, 31]. While the c.2 T>C variant disrupts the translation start codon of *ORC6* transcripts, the presence of nearby downstream in-frame methionine codons (Met20, Met50 and Met59) makes it theoretically possible that an N-terminal truncated protein could be translated, which could harbour some residual functionality. A minigene splicing assay confirmed that the intronic c.449+5G>A variant causes in-frame exon skipping of exon 4, which would result in a translated protein lacking 30 amino acids in the DNA binding domain (Fig. 1) [31].

Using *Drosophila* as a model, the introduction of the patient variant, p.Tyr232Ser (p.Tyr225Ser in *Drosophila*), caused impaired ORC formation by interrupting the interaction of orc6 with orc3, which was then further validated using human ORC6 in vitro [28]. Reducing orc6 levels using a knockdown model also showed that variants orthologous to p.Tyr232Ser and p.Lys23Glu were not able to rescue the mutant phenotype, the former impairing complex formation, and the latter impairing DNA binding. Both variants noticeably reduced pre-RC formation and DNA replication [27, 32].

### CDT1

Variants in *CDT1* appear to be the most common cause of MGORS, and much like *ORC1* individuals, there is a spectrum of clinical presentation [19, 33]. Successful pregnancies have been noted in an adult case with MGORS but the female patient did experience complications requiring a hysterectomy [33]. While adult cases of MGORS known to the clinical genetics community are rare, several females have noted abnormalities of the reproductive tract including the uterus, suggesting this could be an under-reported issue for women with MGORS.

The most common variant seen in *CDT1* is p.Arg462Gln, reported in 11 individuals in the literature so far. It is most often observed in combination with more disruptive variants such as premature stop variants [3, 7, 34]. A mouse model studying the orthologous variant (p.Arg474Gln) discovered that this variant partially impairs MCM binding [35], which may disrupt the essential role of CDT1 in MCM2-7 recruitment to origins of replication.

CDT1 patient substitutions including p.Arg462Gln were analysed in vitro [36]. Substitutions p.Arg462Gln and p.Glu468Lys disrupted the protein surface required for MCM6 binding, resulting in less efficient recruitment of MCM proteins to start sites. One of the more surprising results of this study was the discovery that the p.Ala66Thr variant acts in a hypermorphic manner, rather than hypomorphic as expected. This variant did not reduce stability of CDT1 and was consistently seen to induce increased re-replication compared to other variants and the wildtype control. Despite this, increased CHK1 phosphorylation in the presence of this variant strongly reduced proliferation in a colony-forming assay, suggesting the overall cellular consequence would still be reduced proliferation [36]. While this variant does not appear to impact MCM interactions, it is predicted to impair the binding of the inhibitor molecule cyclin A. This does not impair the nearby CDK-mediated phosphorylation and subsequent binding of SCF E3 ligase skp2 for degradation but nevertheless appears to act through a different, unidentified checkpoint in S or G2 phase to reduce overall proliferation [36].

### CDC6

The first MGORS patient reported was homozygous for a missense variant located within the AAA+ ATPase domain in the encoded CDC6 protein [3]. Such a substitution would likely impact ATPase activity, hampering the ability of CDC6 to disengage from ORC as part of pre-RC assembly [37, 38]. The second patient reported was compound heterozygous for a nonsense and a missense variant [39]. In addition to the characteristic features of MGORS, this second individual was severely affected, with features of progeroid appearance at birth and lipodystrophy; features also observed in one individual with *MCM7* variants [8], and in other replisome disorders affecting later stages of DNA replication initiation [13]. The patient had significant short stature and no microcephaly but did present with motor development delays. The residue affected by the substitution, Lys77, lies within a CDK2/cyclin A phosphorylation motif associated with cytoplasmic translocation to regulate CDC6 activity [39].

Both knockout and hypomorph *cdc6* zebrafish models have been studied [40]. While the knockout was early embryonic lethal, the hypomorph model showed a similar growth profile to the individuals with *CDC6* variants. Interestingly, male zebrafish had a shorter life span as well as reproductive defects, but such comparisons with affected individuals are not yet possible given the rarity and age of the *CDC6* cohort.

### GMNN

Pathogenic de novo variants in three individuals have been identified in *GMNN* [5], which encodes the negative regulator of DNA replication, GEMININ. Affected individuals had been previously clinically diagnosed with MGORS, with all possessing the classical triad of features. Microcephaly and genital anomalies were more variable (each recorded present in only one case), and there was some evidence of developmental delay (2/3 cases) (Table 1).

This is the only described example of an autosomal dominant inheritance pattern of MGORS, and the variants act through a specific mechanism to reduce DNA replication initiation. The N-terminal portion of GEMININ contains a destruction box (residues 23–31) similar to those found in mitotic cyclins [41]. APC ubiquitylation recognises this motif and signals for GEMININ degradation during late M/early G1 phase, thereby enabling the target of GEMININ, CDT1, to be active and assist the loading of MCM helicases on to chromatin [41, 42]. All three variants identified in *GMNN* lie near the 5′ end of the transcript. These disrupt translation of the full protein and either introduce a premature stop codon or are predicted to alter splicing of the first coding exon of *GMNN*. Despite the premature stop codons, the transcripts remained stable, with in vitro overexpression assays showing production of a smaller molecular weight product which is predicted to be translated from a downstream methionine (Met28). Given this protein translation starts within the destruction box motif, such a control mechanism for GEMININ degradation would not be active, ultimately causing less CDT1 activity and a similar effect as other MGORS genes on DNA replication initiation [5].

### MCM3

Recently, a single individual from a consanguineous family has been described as homozygous for a missense variant in *MCM3* [8]. The affected individual possessed some of the cardinal features of MGORS, including the facial gestalt and microtia, along with a proportionate reduction in growth, but patellae were reported as normal. She also had congenital lobar pulmonary emphysema. Despite functional investigations studying the variant in patient-derived cells, no effects on MCM3 transcript or protein levels, or on cell cycle progression could be demonstrated. The variant remains a strong candidate based on it’s location in the C-terminal domain, which is required in chromatin loading of the MCM complex [43], as well as supportive evidence through the identification of pathogenic variants in other MCM subunits in MGORS individuals.

### MCM5

Only one family has been described thus far segregating variants in *MCM5* [12]. The affected boy was biallelic for a frameshift and a missense variant in *MCM5* and displayed typical MGORS features of short stature, microtia, patella agenesis, and bilateral cryptorchidism (Table 1). The individual had normocephaly, and unilateral kidney hypoplasia which likely caused recurrent intestinal infections. The authors studying this patient undertook a comprehensive suite of experiments, confirming a reduction of MCM5 protein in patient-derived cells in both whole-cell extracts and at chromatin, along with a concomitant reduction in MCM2 at chromatin [12]. These cells were slower to progress through S-phase but did not show an increase in replication stress nor any centrosome abnormalities. The missense variant, p.Thr466Ile, lies within the AAA+ ATPase domain of MCM5 (Fig. 1), and mutation of this residue in a yeast complementation assay reduced survival. Previously, zebrafish *mcm5* mutants have been studied and showed a similar overall growth reduction in the absence of any other significant developmental anomalies [4, 44].

### MCM7

MCM7 has recently been implicated in a broad range of phenotypes known to be associated with disorders of DNA replication [8, 45]. Three families have been described in the literature to date; an adult patient from one family has the classic MGORS features of short stature, microtia, and bilateral absent patella. In addition, she had proportional microcephaly and showed absence of breast tissue development (Table 1). She was compound heterozygous for nonsense and missense variants in *MCM7*. The second case showed a similar genotype, although the missense variant affected a residue in a different domain of MCM7. His set of clinical features was quite different, with normal growth parameters but a progeroid appearance at birth, as well as adrenal insufficiency and lipodystrophy – similar features have been observed in cases with *CDC6* or *POLD1* variants [13, 39]. In both cases, patient-derived cells showed a reduction of MCM7 protein, sufficient to cause a mild reduction in DNA replication in early S phase. In vitro studies of the missense variants confirmed that both impacted interactions within the MCM complex [8]. Most recently, a consanguineous family was described segregating a homozygous missense variant in *MCM7* that causes mild microcephaly, short stature, and severe intellectual disability [45]. Interestingly, the missense variant in this third family was located close to the missense variant in the second case described, but the patients show quite different sets of clinical features. The MCM complex serves two roles in DNA replication: firstly, once activated it unwinds DNA and permits entry for the polymerases and other replisome components, and then secondly, the complex functions to continue this unwinding along the chromosome, to help ensure efficient DNA replication. Given the nonsense variants will elicit nonsense-mediated decay (causing null alleles), the position of the residues affected by the substitutions likely plays a role in causing different impacts to MCM7 functioning, and potentially leads to different phenotypes. Further investigation into the different consequences of these patient-informed substitutions could provide further insight.

### CDC45

Variants in *CDC45* underlie a MGORS subtype clinically characterised by the very common appearance of craniosynostosis [6, 46–48]. Outside of the *CDC45* MGORS cohort, craniosynostosis has only been observed in two other MGORS individuals, one with *ORC1* variants and another recently identified homozygous for a *GINS2* variant (Table 1) [4, 11]. The strong association of craniosynostosis with *CDC45* variants supports the use of this feature as a differential diagnosis in genetic testing. Identification of biallelic *CDC45* variants in a MGORS cohort was the first evidence for the involvement of the pre-initiation and CMG helicase complexes in the pathogenesis of MGORS [6].

To date, biallelic *CDC45* variants have been reported in 19 individuals across 15 families and underlie a broad spectrum of phenotypes that range from a classic MGORS presentation to syndromic craniosynostosis with short stature but no other MGORS features [6, 46–48]. The majority of clinically diagnosed MGORS individuals had the clinical triad of short stature, microtia, and patella hypo/aplasia [37–39]. MGORS-related genital anomalies (micropenis or clitoromegaly) were reported in at least two individuals and both females of suitable age were affected by mammary hypoplasia [37]. Craniosynostosis was present in many of the individuals with *CDC45* variants but is an incompletely penetrant trait (13/14 individuals), as observed by the discordance of bicoronal craniosynostosis in two affected brothers (Table 1) [6]. Anorectal malformations are common and range from anal stenosis to an imperforate or anteriorly placed anus. Heart and palate malformations are occasionally present (7/13 and 2/13 cases respectively), and sparse eyebrows are invariant amongst all individuals with *CDC45* variants. *CDC45* is located on chromosome 22, within the genomic region associated with DiGeorge syndrome (MIM 188400). Sequencing of a cohort of individuals with DiGeorge syndrome revealed several hemizygous variants in *CDC45* in those with atypical features similar to the MGORS cohort described [49]. Functional assays will be required to confirm the consequence of these variants on CDC45 levels and function and determine their involvement in the phenotypes presented, given some are found in a homozygous state in control populations, or lie within non-canonical or non-coding exons.

Across the *CDC45* cohort there are a range of truncating, non-synonymous, and synonymous splice-altering variants but no biallelic truncating genotypes were reported. Missense variants do not cluster within a particular domain nor with a particular phenotype, but many are found in the conserved core region of CDC45 and are expected to destabilise the protein. In keeping with these predictions, cells derived from several patients demonstrated reduced CDC45 levels [6].

Interestingly, synonymous splice-altering variants appear to be more frequently found in the *CDC45* cohort. These include variants c.318C>T and c.333C>T that are present in patients with syndromic craniosynostosis and the MGORS-subtype respectively. Both splicing variants cause the same effect of increased skipping of exon 4. The exclusion of this exon keeps the transcript in-frame and is observed at low levels in unaffected cells. However, since this removes part of the conserved DHH phosphoesterase domain, its presence is unexpected. A third synonymous splice-altering variant, c.630G>A, was found in the non-canonical exon 7 (numbering based on RefSeq: NM_001178010.2) in siblings with MGORS with craniosynostosis [46]. The splicing variant both reduced the level of canonical transcript and produced three alternative transcripts, all of which were expected to undergo nonsense-mediated decay. A fourth synonymous variant, c.1512C>T, has also been identified in MGORS, but functional analysis has been not undertaken to assess the impact on canonical splicing [47].

### GINS2

Only one *GINS2*-associated case has been reported thus far, who is homozygous for a missense variant (p.Arg114Leu) [11]. In addition to the characteristic triad, this patient has many features in common with the CDC45-MGORS subtype, such as craniosynostosis, atrial septal defect, and an anteriorly placed anus (Table 1). The orthologous missense variant shows defects in a yeast assay of DNA replication, and the resolved CMG helicase structure places this residue at the interaction site between GINS2, CDC45, and MCM5 [11, 50].

### GINS3

Recently, biallelic missense variants in *GINS3* were also reported to underlie MGORS [10]. All affected individuals possessed at least one variant affecting p.Asp24, which was commonly substituted to glycine or less often, asparagine. Extensive analysis showed these variants impact on the protein stability of GINS3. Mutant GINS3 is reduced at chromatin, which has a broader effect on other members of the GINS complex and other protein components of the replisome. These effects then impact on replication speed, interorigin distance, and S phase length, ultimately slowing proliferation. Interestingly, unlike human counterparts homozygous for the same variant, the mouse model harbouring the p.Asp24Asn variant was embryonic lethal with fetal resorption starting from E16.5 [10]. Embryonic fibroblasts derived from E12.5 mutant embryos show premature senescence, which could potentially be due to reduced DNA replication.

The patient cohort demonstrated the characteristic features of MGORS, with a broad spectrum of other features (Table 1) [10]. All individuals with medical information demonstrated microcephaly (6/6 cases), but not all had short stature (6/7 cases). The growth restriction was not proportionate in many individuals, and while some had a more severe height restriction, others had a smaller head circumference. The facial gestalt common in MGORS was not so apparent in this cohort, although all had some degree of microtia or low-set ears. Patella agenesis was very common, but not universal (3/4 cases). Interestingly, many individuals showed neutropenia (4/5 cases), with one also showing B lymphopenia. Given the unique overlap of MGORS and neutropenia in *GINS3* individuals, these features could be used diagnostically as differentials in candidate gene testing.

### DONSON

Exome sequencing and phased genome sequencing identified *DONSON* as a novel disease gene in several MGORS patients [9, 16, 17]. DONSON is a replisome component and replication fork stabiliser that is involved in activating the ATR-dependent replication stress response [15]. *DONSON* is unique compared to all other MGORS genes as the encoded protein has no known role in either the DNA pre-RC or pre-IC [15].

All of the DONSON-MGORS cohort had short stature, microtia and patella agenesis (Table 1) [9, 16, 51]. Most had proportionate microcephaly, but one patient had an average head circumference for their age [9]. In vitro analysis of MGORS patient variants demonstrated that the substitutions reduced nuclear localisation of DONSON, and a deep intronic variant introduces a novel strong splice acceptor site causing a reading frameshift and premature stop codon, confirming the hypomorphic nature of these variants [9].

Biallelic hypomorphic *DONSON* variants have previously been found to underlie a clinically distinct primordial dwarfism of severely disproportionate microcephaly and variable skeletal abnormalities mainly present in the upper limb (MISSLA; MIM 617604) [15, 51, 52]. A third associated phenotype is microcephalic-micromelia syndrome (MIMIS; MIM 251230), a severe form of primordial dwarfism associated with perinatal death [53, 54]. Microcephaly in MIMIS patients is severe but proportional to height restriction, with the identified MIMIS variants causing a severe or predicted complete loss of DONSON protein. Given the early embryonic lethality of a mouse knockout model [54], a rescue via an alternative transcript could be possible to explain the extended survival in MIMIS individuals.

The most pronounced phenotypic difference between DONSON-MGORS and DONSON-MISSLA individuals is the degree of severity of microcephaly versus short stature. DONSON-MISSLA exhibit a more severe microcephaly compared to height reduction, whereas these parameters are much more proportionate in DONSON-MGORS individuals [9]. Interestingly, like *DONSON*, pathogenic variants in multiple other genes encoding proteins also involved in replication fork stabilisation or the ATR-dependent stress response pathways cause a phenotypically similar primordial dwarfism, Seckel syndrome, that also features severely disproportionate microcephaly [13]. Some patients in the DONSON-MISSLA cohort exhibit phenotypic similarity to MGORS with patella hypoplasia and microtia present variably, for example, patient P19 from the original MISSLA cohort demonstrates a phenotype potentially better aligned with MGORS than with the rest of the MISSLA cohort [15]. This male patient is homozygous for p.Pro433Ser, a genotype independently reported in a different patient clinically diagnosed with MGORS [17]. Patient P19 had characteristic MGORS features of microtia, proportionate short stature, and microcephaly. While patella hypo/aplasia was not noted for this individual, mild genital hypoplasia was present, which is common in MGORS [20]. Three further patients from the initial MISSLA cohort (P9 and brothers P20-1 and P20-2) also show phenotypic overlap with MGORS. They each meet the diagnostic criteria for MGORS with microtia and patella hypo/aplasia, and brothers P20-1 and P20-2 also have cryptorchidism [15]. While the growth parameters of these cases are more in keeping with MISSLA, their skeletal anomalies are minor compared to the rest of the MISSLA cohort and restricted to fingers, toes, and metacarpals. Together, such cases highlight the complexity of reconciling a clinical and genetic diagnosis involving *DONSON*.

**Fig. 2:**
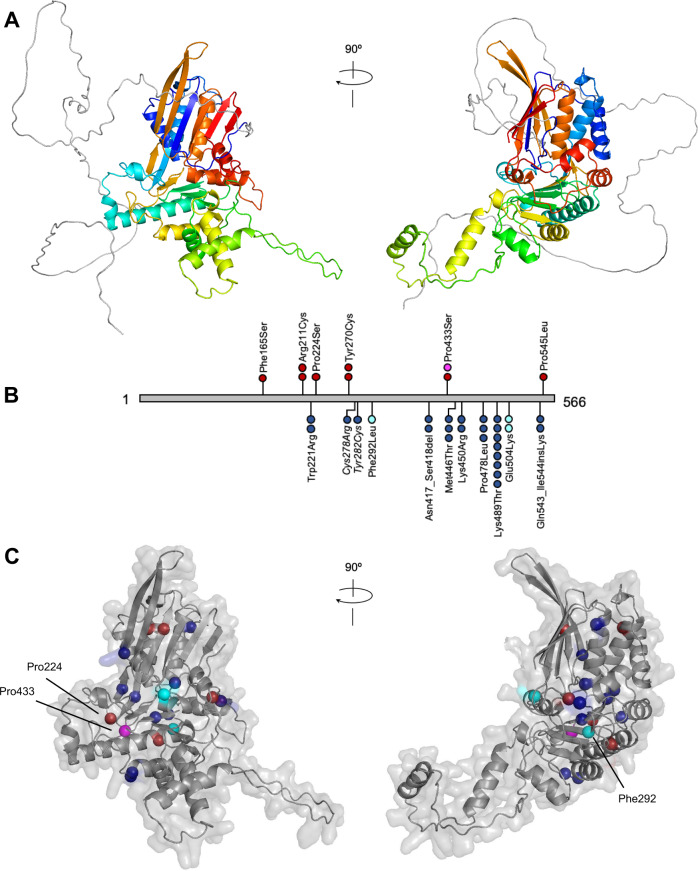
Predicted structure of human DONSON and the locations of DONSON-MGORS and MISSLA missense variants. **A** Predicted structure of human DONSON (AlphaFoldDB ID: DONS_HUMAN). DONSON is composed of a 150-residue long intrinsically disordered N-terminal region (grey) followed by interfolded protein (residues 151-566) coloured rainbow from the N-terminal end (blue) to the C-terminal end (red). **B** Linear DONSON (grey) showing the location of missense variants found in MGORS (above) and MISSLA (below). Each sphere represents one individual: red - MGORS, blue - MISSLA, magenta - MISSLA individual with clear MGORS features, cyan - MISSLA patients with phenotypic overlap to MGORS. Variants Cys278Arg/Tyr282Cys are italicized as either one, or both, are pathogenic in a single individual. **C** Position of MGORS and MISSLA-affected residues within the predicted DONSON structure. Residues are coloured by patient phenotype as per **B**. Indicated residues are discussed further in the main text. No variants are in the 150-residue-long intrinsically disordered N-terminal region and so for clarity it was removed.

While the role of DONSON in DNA replication and cell cycle progression has become clearer [15, 55, 56], the protein itself is somewhat of an enigma. There are no predicted protein domains nor is it similar to any other known protein. With the recent release of AlphaFold [57, 58], we have been able to view a possible structure for DONSON for the first time. The AlphaFold prediction (AlphaFoldDB ID: HUMAN_DONS) (Fig. 2A) suggests an intrinsically disordered N-terminal region of approximately 150 amino acids, ahead of one interconnected domain that is joined together via a less ordered looped structure. This interconnected domain is predicted by AlphaFold to have a confident/very high model confidence with a pLDDT score of >70/>90.

MGORS missense variants are spread throughout the DONSON protein sequence and do not cluster together in the linear protein sequence nor in the predicted 3D structure (Fig. 2B, C) [9]. MISSLA missense variants are also spread throughout the linear protein sequence, although they tend to lie towards the C-terminus. Most residues substituted in MISSLA are scattered throughout the 3D structure. Four of them are located within the core anti-parallel β-sheet, including Phe292, which is found in a patient with phenotypic overlap with MGORS. Although no MGORS variants are located within this core β-sheet structure, two proline residues that are substituted in MGORS, Pro224, and Pro433, are each found at the flank of a β-strand involved in this β-sheet. Further functional analysis will shed light on the role of this core in DONSON functioning.

From the evidence currently available, there is no obvious genotypic/phenotypic relationship between MGORS and MISSLA patients. While both syndromes are caused by reduced function, specific variants or genotypes have not been compared to understand the relative severity on protein activity or function, such as their effect on protein-protein interactions.

The involvement of a replication fork stabilisation factor in MGORS potentially challenges MGORS as being the consequence of disrupted early-replication organisation. MGORS could therefore be considered to be a disorder of broader DNA replication processes including late-stage replisome stability, or alternatively DONSON may have additional unknown roles in early replication processes. Given that new complexities of DNA replication initiation are still being uncovered [59], and the recent confirmation of DONSON as a DNA replication protein, the latter remains a possibility. Further investigation into DONSON domains and function could help to clarify whether there are genotypic/phenotypic relationships between the clinically distinct syndromes.

## Non-canonical functions of MGORS-associated proteins

A number of other cellular functions have been described for proteins linked to MGORS. For example, ORC6 acts to promote cell abscission during cytokinesis and also has a role in mismatch repair during DNA synthesis [60, 61], while CDT1 enables stable kinetochore-microtubule interactions during mitosis [62, 63]. Non-canonical functions of some of the encoded proteins include the presence at the centrosome or cilium (reviewed in ref. [64]). There is accumulating evidence supporting ORC and MCM complexes at the centrosome [65], with this organelle serving as a possible hub for cell cycle progression interactions, such as ORC1 with cyclin E [66]. ORC1 assists in regulating centriole and centrosome duplication via interaction of two separate domains. The cyclin-CDK2 binding domain contains MGORS variants; p.Arg105Gln entirely abolishes inhibition of cyclin E-CDK2, while p.Phe89Ser and p.Glu127Gly partially impede inhibition of both cyclin A-CDK2 and cyclin E-CDK2. The result of abolished Cyclin-CDK inhibition is centrosome reduplication and loss of centrosome copy number control [67].

Cilia defects have been observed at a low level in MGORS patient-derived cells, or when MGORS proteins are subjected to siRNA in reporter cell models [64]. Zebrafish models, using morpholinos targeting some of the MGORS proteins, show some phenotypes that overlap with those observed in zebrafish models of ciliopathies. In these models, rescuing with a patient-informed mutant mRNA did not rescue the ciliopathic phenotypes, but wildtype mRNA also only resulted in a partial phenotypic rescue. Based on these observations, a hypothesis has been put forward that MGORS could be considered a ciliopathy [64, 68, 69]. Such a suggestion needs to be considered with care, for both clinical and biological reasons. Clinically, ciliopathies are distinguished by characteristic features such as retinal dystrophy, polydactyly, cystic kidneys, or obesity [70] requiring specific clinical management, which have never been observed in MGORS individuals [2, 19]. Furthermore, while many of the ORC and MCM subunits have been found localised at the centrosome, not all MGORS-associated proteins have, such as CDC45, or proteins linked to MGORS-like clinical features, such as RECQL4 or POLE. The presence of these proteins at the centrosome is intriguing, and suggests complex biology linking DNA replication, cell cycle progression and centrosome duplication and ciliogenesis, but the pathognomonic relevance to MGORS is less certain. Given the long list of MGORS associated genes identified as highlighted in this review, which account for a majority of affected individuals, the most parsimonious explanation remains that MGORS is a disorder of DNA replication.

## The expanding spectrum of disorders linked to DNA replication

While the MGORS variants in the genes encoding the pre-RC were amongst the first germline variants to be described involving DNA replication, since then, many components in this complex process have become linked to genetic disorders [13]. It is common for these syndromes to be characterised by growth restriction, microcephaly and/or short stature. As further individuals and more disease-causing genes are identified, some interesting patterns are appearing which give tantalising clues to the underlying developmental biology disrupted by such variants. A key example is effects on haematopoiesis. Neutropenia has not been described as a common feature associated with any other MGORS gene before, with *GINS3* being the first description of such an overlap. However, neutropenia is commonly associated with variants in other GINS subunits, *GINS1* and *GINS4* [71, 72], and in at least some individuals with a biallelic variant in the functionally related subunit of the MCM complex, *MCM4*, but not *GINS2*, *GINS3* or *MCM3*, *MCM5* or *MCM7* [73]. These clinical findings in *GINS3* adds to the accumulating evidence for the role of a sub-section of replication initiation factors in haematopoiesis and immune cell functioning [13].

Microtia and patella hypo/aplasia have been considered differential features of MGORS [74], but clinical genetic findings reveals a more complicated picture. These features have also been reported in individuals with variants in genes such as *RECQL4* and *POLE*. Such patients do not have the facial gestalt that is commonly observed in MGORS but these overlaps do indicate a broader link between skeletal and/or cartilage development and efficient organisation and initiation of DNA replication. There is emerging evidence linking DNA replication initiation to cell fate [75, 76], and the wealth of human genetics summarised here adds to this. It will be fascinating to learn how tissue-specific development and the ubiquitous requirement of DNA replication initiation are so intimately linked.

## Conclusion

High-throughput sequencing approaches in clinical genetics have expanded the molecular and clinical understanding of MGORS. As the number of genes associated with MGORS increases, we see the appearance of subtypes, notably the prevalence of craniosynostosis in CDC45-associated MGORS and neutropenia in GINS3-associated MGORS. The classical triad of short stature, microtia, and patella hypo/aplasia that historically defined MGORS is more variable than previously thought. Conversely, mammary hypoplasia is the most consistent feature observed in MGORS, and external genital abnormalities and skeletal anomalies are also common. While gene discoveries continue to provide insights into MGORS and the link between DNA replication and human development, unique individuals illustrate the clinical complexities of a multi-gene disorder such as MGORS.
